# On the development of modular polyurethane-based bioelastomers for rapid hemostasis and wound healing

**DOI:** 10.1093/rb/rbad019

**Published:** 2023-03-09

**Authors:** Wanxin Guo, Binan Zhao, Muhammad Shafiq, Xiao Yu, Yihong Shen, Jie Cui, Yujie Chen, Pengfei Cai, Zhengchao Yuan, Mohamed EL-Newehy, Hany EL-Hamshary, Yosry Morsi, Binbin Sun, Jianfeng Pan, Xiumei Mo

**Affiliations:** State Key Laboratory for Modification of Chemical Fibers and Polymer Materials, Shanghai Engineering Research Center of Nano-Biomaterials and Regenerative Medicine, College of Biological Science and Medical Engineering, Donghua University, Songjiang, Shanghai 201620, P.R. China; Department of Orthopedics, Shanghai Tongji Hospital, School of Medicine, Tongji University, Putuo, Shanghai 200065, China; Department of Chemical Engineering, Faculty of Engineering, Graduate School, Kyushu University, Fukuoka 819-0395, Japan; State Key Laboratory for Modification of Chemical Fibers and Polymer Materials, Shanghai Engineering Research Center of Nano-Biomaterials and Regenerative Medicine, College of Biological Science and Medical Engineering, Donghua University, Songjiang, Shanghai 201620, P.R. China; State Key Laboratory for Modification of Chemical Fibers and Polymer Materials, Shanghai Engineering Research Center of Nano-Biomaterials and Regenerative Medicine, College of Biological Science and Medical Engineering, Donghua University, Songjiang, Shanghai 201620, P.R. China; State Key Laboratory for Modification of Chemical Fibers and Polymer Materials, Shanghai Engineering Research Center of Nano-Biomaterials and Regenerative Medicine, College of Biological Science and Medical Engineering, Donghua University, Songjiang, Shanghai 201620, P.R. China; State Key Laboratory for Modification of Chemical Fibers and Polymer Materials, Shanghai Engineering Research Center of Nano-Biomaterials and Regenerative Medicine, College of Biological Science and Medical Engineering, Donghua University, Songjiang, Shanghai 201620, P.R. China; State Key Laboratory for Modification of Chemical Fibers and Polymer Materials, Shanghai Engineering Research Center of Nano-Biomaterials and Regenerative Medicine, College of Biological Science and Medical Engineering, Donghua University, Songjiang, Shanghai 201620, P.R. China; State Key Laboratory for Modification of Chemical Fibers and Polymer Materials, Shanghai Engineering Research Center of Nano-Biomaterials and Regenerative Medicine, College of Biological Science and Medical Engineering, Donghua University, Songjiang, Shanghai 201620, P.R. China; Department of Chemistry, College of Science, King Saud University, Riyadh 11451, Saudi Arabia; Department of Chemistry, College of Science, King Saud University, Riyadh 11451, Saudi Arabia; Faculty of Engineering and Industrial Sciences, Swinburne University of Technology, Boroondara, VIC 3122, Australia; State Key Laboratory for Modification of Chemical Fibers and Polymer Materials, Shanghai Engineering Research Center of Nano-Biomaterials and Regenerative Medicine, College of Biological Science and Medical Engineering, Donghua University, Songjiang, Shanghai 201620, P.R. China; Department of Orthopedics, Shanghai Tenth People’s Hospital, School of Medicine, Tongji University, Jingan, Shanghai 200072, China; State Key Laboratory for Modification of Chemical Fibers and Polymer Materials, Shanghai Engineering Research Center of Nano-Biomaterials and Regenerative Medicine, College of Biological Science and Medical Engineering, Donghua University, Songjiang, Shanghai 201620, P.R. China

**Keywords:** polyurethane, electrospinning, hemostasis material, wound healing, tissue regeneration, regenerative medicine

## Abstract

Massive hemorrhage may be detrimental to the patients, which necessitates the advent of new materials with high hemostatic efficiency and good biocompatibility. The objective of this research was to screen for the effect of the different types of bio-elastomers as hemostatic dressings. 3D loose nanofiber sponges were prepared; PU-TA/Gel showed promising potential. Polyurethane (PU) was synthesized and electrospun to afford porous sponges, which were crosslinked with glutaraldehyde (GA). FTIR and ^1^H-NMR evidenced the successful synthesis of PU. The prepared PU-TA/Gel sponge had the highest porosity and water absorption ratio. Besides, PU-TA/Gel sponges exhibited cytocompatibility, negligible hemolysis and the shortest clotting time. PU-TA/Gel sponge rapidly induced stable blood clots with shorter hemostasis time and less bleeding volume in a liver injury model in rats. Intriguingly, PU-TA/Gel sponges also induced good skin regeneration in a full-thickness excisional defect model as revealed by the histological analysis. These results showed that the PU-TA/Gel-based sponges may offer an alternative platform for hemostasis and wound healing.

## Graphical Abstract

**Graphical Abstract:**
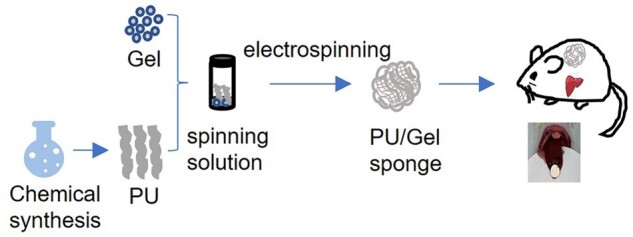

## Introduction

Massive wound bleeding caused by trauma can cause a variety of complications, such as coagulation disorders, inflammation and multiple organ injury, which may increase medical costs, morbidity and mortality [1]. In emergency treatment in the medical field, one of the primary requirements for hemostatic products is to achieve rapid and effective control of bleeding, to save lives, and to have low toxic and side effects on organisms [2]. In addition, in the face of massive wound bleeding, the rapid formation of a blood clot in the wound and blocking the blood flow is an effective treatment. However, this is usually achieved by using hemostatic materials and devices. A lot of systematic studies have been conducted to develop materials for hemostasis and wound healing [3–6]. The ideal hemostatic materials should have the following properties: stop bleeding in <2 min, biocompatible, clinical safety, easy to use and inexpensive, etc [7]. Undoubtedly, it is important to research new hemostatic materials with good hemostatic ability, high efficiency, safety and ready-to-use.

Active chemical composition and material form are important factors to be considered in the development of hemostatic materials [8]. Previous studies mostly used natural polymer-based materials with active components as hemostatic materials to control bleeding, such as collagen [9], gelatin [10], chitosan [11], oxidized cellulose [12], alginate [13], etc. These materials showed excellent hemostatic properties. However, natural polymer materials may have immunogenicity problems due to their animal origin. In contrast, synthetic polymers can avoid these limitations. Different types of synthetic polymers, such as polyurethane (PU), polycaprolactone, polylactic acid and polyvinyl alcohol have been used [14–17]. In addition, hybrids based on natural and synthetic polymers have also been exploited to afford hemostatic biomaterials [18, 19]. The synergistic effect of the hemostatic active components was used to improve the hemostatic performance of the material [2, 20].

Polyurethanes, also known as carbamate, are synthetic polymers consisting of soft and hard segments linked by urethane linkages [21, 22]. The physicochemical properties of the PU can be altered by varying the ratio or composition of hard and soft segments [23]. PU has been widely exploited for biomedical and pharmaceutical applications owing to its low cytotoxicity, high oxygen permeability, good biocompatibility and biodegradability. Besides, PU has been widely used for cardiovascular and musculoskeletal tissue regeneration applications [24–28]. PU has also been used along with other natural polymers, including cellulose and chitosan, thereby further establishing the importance of these hybrids for tissue engineering (TE) applications.

Gelatin (Gel) which is obtained by the denaturation and hydrolysis of collagen is another promising candidate for TE applications, owing to its low-cost, good biocompatibility and biodegradability [29, 30]. Besides gelatin may provide a conducive environment for cytocompatibility and tissue regeneration [31]. Gelatin may also activate platelets, induce platelet aggregation, promote the formation of clots and enhance the formation of thrombin to achieve the purpose of quick hemostasis [32, 33].

While hemostatic biomaterials in different shapes and structures, such as micro/nanoparticles, 2D and 3D structures, hydrogels and fiber sponges have been exploited, the latter exhibit promising potentials [34–36]. Electrospun fibers offer a promising option due to their tunable porosity and extracellular matrix (ECM)-mimetic morphology.

**Scheme 1.**
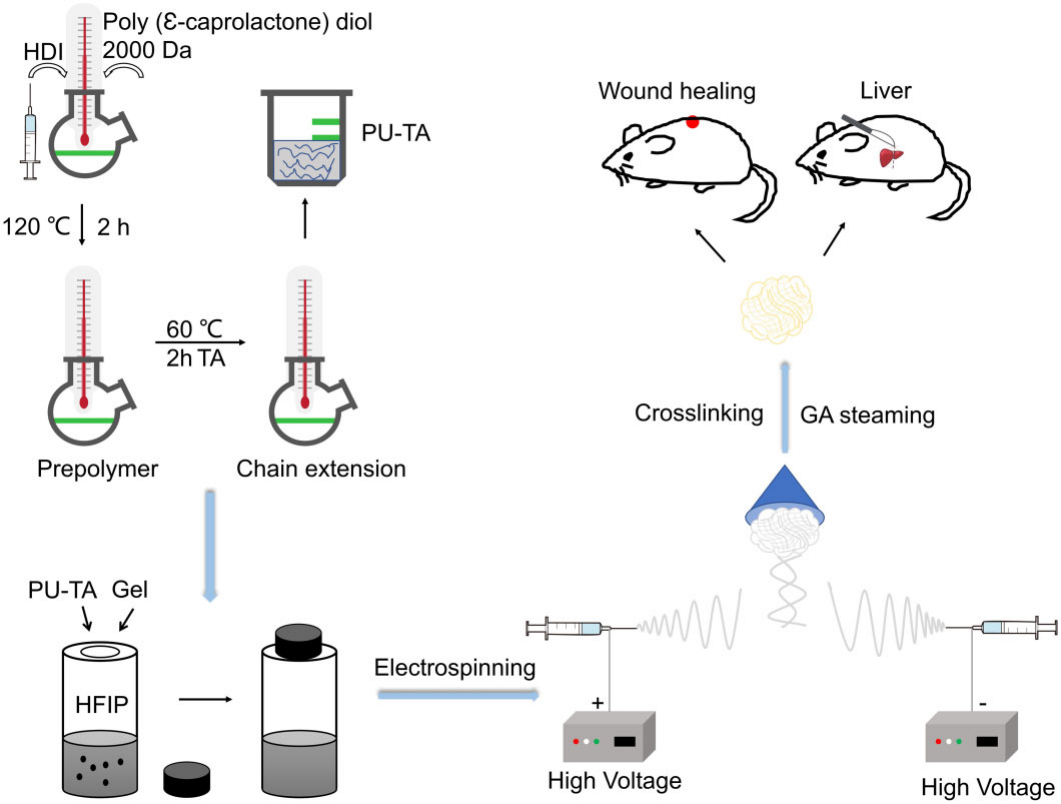
Preparation and application of PU/Gel fiber sponges.

The overarching objective of this study was to synthesize PU by using different types of extenders and to then exploit them to prepare hemostatic fiber sponges. It is worth noting that previously 1,4-butanediol (BDO) and 2,2-Bis (hydroxymethyl) propionic acid (DMPA) have been exploited as chain extenders for the synthesis of PU owing to their carboxylic (–COOH) or hydroxyl (–OH) groups. On the other hand, the molecular structure of tranexamic acid (TA) is similar to lysine, which can competitively inhibit the binding of the plasminogen to the fibrin, thereby suppressing the degradation of fibrin and reducing blood loss by forming clots. Adenosine diphosphate (ADP) may also induce platelet aggregation and promote clotting. Therefore, we exploited traditional extenders, including BDO and DMPA as well as TA and ADP for the synthesis of PU. Unique fibrous structure and high porosity of the sponge may help achieve good hemostatic ability for visceral bleeding and wound healing (Scheme 1). The hemostatic effect of the prepared sponge was evaluated by measuring blood coagulation index, whole blood clotting time, erythrocyte adhesion and prothrombin time (PT)/activated partial thromboplastin time (APTT). Physico-chemical Moreover, hemostatic ability, cytocompatibility and *in vivo* biocompatibility of dressings were assessed.

## Materials and methods

### Materials

Polycaprolactone diol (PCL diol, *M*_w_ = 2000 Da), and gelatin from porcine skin were purchased from Sigma-Aldrich, Shanghai, China. Dimethyl sulfoxide-d6 (DMSO-d6, 99.8%), stannous octoate (Sn(Oct)_2_, purity = 95%), TA (purity = 99%) and 2,2-Bis (hydroxymethyl) propionic acid (DMPA) were purchased from Shanghai Titan Technology Co., Ltd. DMSO (purity = 99.7%) was purchased from Beijing Innochem Science & Technology Co., Ltd, Beijing, China. Hexamethylene diisocyanate (HDI, purity = 98.5%), BDO (purity = 99%) and methylbenzene (purity = 99.5%) were purchased from Guoyao Reagents Company (Shanghai, China). ADP (purity = 98%) was purchased from Beijing Hwrkchemical Co., Ltd., Beijing, China. PT and APTT test kits were purchased from Shanghai Sun Biotechnology Co., Ltd., Shanghai, China. Dulbecco’s Modified Eagle Medium (DMEM) was purchased from Shanghai MesGen Biotechnology Co., Ltd, Shanghai, China. Fetal bovine serum (Gibco) was purchased from Beyotime, Shanghai, China.

### Synthesis of polyurethanes

**Scheme 2.**
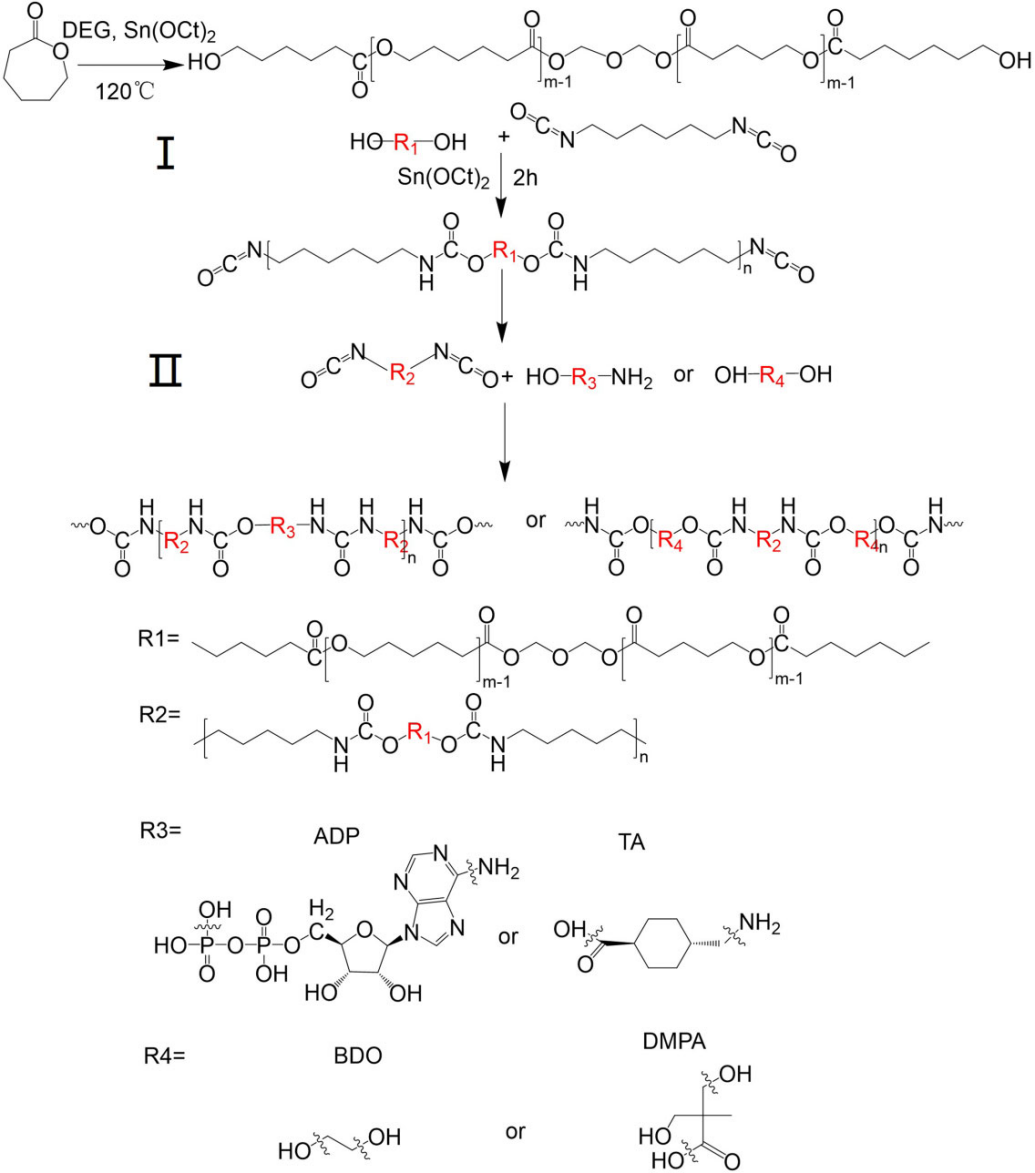
Schematic illustration for the synthesis of PU. (I) Synthesis of prepolymer; (II) reaction of prepolymer with chain extender to prepare high molecular weight polyurethane.

Polyurethane was synthesized by using a two-step solution polymerization method (Scheme 2). The synthesis was carried out in a three-necked flask under a nitrogen atmosphere. The molar ratios of PCL diol, HDI and chain extender (e.g. ADP, BDO, DMPA, TA, etc.) were 1:2:1, respectively. For the synthesis of a prepolymer, a certain amount of PCL-diol was added into a three-neck flask and heated in an oil bath at 120°C. The moisture was concomitantly removed by using a vacuum pump for up to 2 h. Thereafter, HDI and a catalytic amount of Sn(Oct)_2_ (catalyst: reactant = 1:1000) were added by using a syringe and the temperature was reduced to 60°C for 2 h. Subsequently, a solution of the chain extender (e.g. ADP, BDO, DMPA, TA, etc.) was added to the prepolymer solution for initiating the chain extension. The obtained polymer was collected by precipitation in distilled water, filtered and freeze-dried. The PU was named as PU-ADP, PU-BDO, PU-DMPA or PU-TA according to the type of the used chain extender, respectively.

### Characterization

#### Structural analysis

Structural analysis of polymers was carried out by using Fourier transform infrared spectroscopy (FT-IR) with attenuated total reflection FTIR spectrophotometer (Nicolet AVATAR 380, Thermo Fisher Scientific, USA). The spectra were collected in the range of 4000–400 cm^−1^.

#### Proton nuclear magnetic resonance

Polymers were further analyzed by ^1^H-NMR (AVANCE-400, Bruker company, Switzerland) by using DMSO-d6 as a solvent. The chemical shift was measured in ppm.

### Fabrication of electrospun fibers

PU/gelatin (PU/Gel) fibers were prepared by electrospinning. A certain amount of PU-ADP, PU-BDO, PU-DMPA and PU-TA was weighed and mixed along with Gel according to a specified mass ratio to afford 10% (w/v) solution in hexafluoroisopropanol. The specifications of electrospinning equipment were described elsewhere [37]. Following electrospinning parameters were used for the fabrication of fibers: flow rate, 1.2 ml/h, applied voltage 8 kV, spinneret-to-collector distance, 12 cm, the rotational speed of the drum collector, ∼4 rotations per minute (rpm) and electrospinning time, 4 h. The collected fiber sponge was dried in an oven at 37°C for 48 h and crosslinked by exposing to glutaraldehyde (GA) vapors for 1 h. The fiber sponge was named as PU-ADP/Gel, PU-BDO/Gel, PU-DMPA/Gel or PU-TA/Gel according to the used type of PU.

### Scanning electron microscopy

The morphological analysis of uncrosslinked or crosslinked fibers was performed by scanning electron microscopy (SEM, Phenom XL, Phenom Scientific Instruments Co. Ltd., Shanghai, China) at an accelerated voltage of 10 kV. The average diameter of the cross-linked sponge fibers was measured by ImageJ and was analyzed by Origin 85 software.

### Porosity

The porosity of the fiber sponge was measured following the reported method [38]. First, ethanol was added to the measuring cylinder and the volume was measured as (*V*_1_). Then, the sponge was immersed into the solution until the liquid was absorbed by the sponge; the volume was measured as (*V*_2_). The sponge was removed from the ethanol solution and the volume of the remaining solution was recorded as (*V*_3_). At least three independent samples were evaluated for porosity. The porosity (*P*) of the sponge was calculated by Equation (1):


(1) $$P\;(\%)\;=\frac{V_{1-}V_{3}}{V_{2-}V_{3}}\times 100\%.$$

### Water absorption capacity

The water absorption capacity of the nanofiber sponge was measured by following a previous report with a slight modification [39]. A certain weight of the sponge (*n *=* *3) was measured (*W_d_*). The sponge was placed into a culture dish prefilled with deionized water. The excess water on the surface of the sponge was absorbed and the weight of the wet sponge was recorded (*W_w_*). The water absorption capacity (*W*) of the fiber sponge was calculated by Equation (2):


(2) $$W\;(\%)\;=\frac{W_{w}-W_{d}}{W_{d}}\times 100\%,$$

where *W_d_* and *W_w_* indicate the weight of the dry and wet sponges, respectively.

### *In vitro* degradation

*In vitro* degradation of the sponges was carried out in an aseptic phosphate-buffered solution (PBS). The sponge samples (*n *=* *3) were immersed in polypropylene (PP) tubes containing 2 ml PBS. Tubes were placed into a constant temperature shaker at 37°C. At a specified time point (*t*), samples were removed from the tubes, washed with deionized water, freeze-dried and weighted. The degradation rate (DR) of the fiber sponge was calculated by Equation (3): where *W*_1_ and *W_t_* indicate the weight of the sponge before and after degradation, respectively.


(3) $$\text{DR}=\left(\frac{W_{t}}{W_{1}}\times 100\right)\%,$$

### *In vitro* hemostatic performance

#### Blood clotting index

The blood clotting index (BCI) of the fiber sponge was measured following the reported method [40]. Circular-shaped sponge (diameter = 9 mm) was placed into a petri dish and preheated for 10 min at 37°C. About 100 μl of sodium citrate anticoagulated rabbit blood and 20 μl of CaCl_2_ solution (0.2 M) were dropped onto the sponge and incubated for 1, 3, 5 and 10 min at 37°C. Thereafter, sponges were washed with 10 ml of deionized water to wash away excess blood without affecting the clot. The absorbance of the unclotted blood solution was measured at 540 nm by using a microplate reader (Multiskan MK3, Thermo Fisher Scientific, USA) (*n *=* *6). The gauze was treated in a similar fashion. About 100 μl of sodium citrate anticoagulated rabbit blood diluted with 10 ml deionized water served as a negative control. The BCI was calculated by Equation (4): where *I_s_* and *I_c_* represent the absorbance of the sample and negative control, respectively.


(4) $$\mathrm{BCI}\;(\%)=\frac{I_{s}}{I_{c}}\times 100\%,$$

#### Whole blood clotting time

The test tube inversion method was used to test the whole blood coagulation of the fiber sponge. The gauze was used as a control group. The sponge (weight = ∼10 mg) was added to the PP tube. About 1 ml of whole blood and 60 μl of 0.2 M CaCl_2_ were added into the tube. The tube was inverted every 30 s, and the blood coagulation of the sponge was observed.

#### Erythrocyte adhesion

Sodium citrate anticoagulated rabbit blood was centrifuged at 1300 rpm for 10 min to collect erythrocytes. Erythrocytes were diluted with 0.9% normal saline. Fiber sponge was placed into a 48-well plate and sterilized by ultraviolet (UV) irradiation for 5 min which was followed by the addition of 200 μl of erythrocyte suspension and incubation in a shaking water bath at 37°C for 2 h. Unadhered erythrocytes were washed away with PBS and fixed with paraformaldehyde at 4°C for 2 h. The sponge sample was dehydrated with gradient ethanol series (30%, 50%, 70%, 80%, 90% and 100%) for 10 min, and dried in a vacuum hood. Samples were gold-sputtered and the morphology and number of erythrocytes on the samples were observed by SEM.

#### Activated partial thromboplastin assay

Sodium citrate anticoagulated rabbit blood was centrifuged at 1300 rpm for 10 min. The supernatant was collected and centrifuged at 3000 rpm for 20 min to obtain platelet-deficient plasma (PPP) for activated partial thromboplastin time (APTT) and PT. The sample was put into a plastic tube and incubated with 100 µl of PPP at 37°C for 3 min. Thereafter, 200 μl of preheated PT reagent was added. The time required for the solidification of the PPP was recorded as PT value. The gauze served as a control group.

Similarly, for APTT assay, the sample was put into a plastic tube and 100 µl of PPP was added. About 100 µl of preheated APTT reagent was added and the tube was incubated at 37°C for 5 min. Finally, 100 μl of preheated CaCl_2_ was added. The time required for the solidification of the material was indicated an APTT value. The gauze was used as a control group.

### *In vitro* biocompatibility assay

#### Hemolysis assay

The hemolysis ratio of the fiber sponge was measured following the reported method [41]. Sodium citrate anticoagulated rabbit blood was centrifuged at 1300 rpm for 10 min to collect erythrocytes for hemolysis assay. About 2 ml of erythrocyte suspension was diluted with 2.5 ml of normal saline to afford erythrocyte suspension. Erythrocytes suspension was added into a centrifuge tube into which 200 µl of diluted blood and 10 ml of normal saline were added and the tube was incubated for 2 h at 37° C. About 200 μl of diluted blood admixed with 10 ml deionized water or 10 ml of normal saline served as a positive and negative control, respectively. After incubation, the suspension was centrifuged at 3000 rpm for 5 min. The supernatant was collected and the OD was recorded at 540 nm by using a microplate reader. The hemolysis ratio (HR) of the fiber sponge was calculated by Equation (5): where *A_e_* represents the absorbance of the sample, *A_p_* refers to the positive group and *A_n_* denotes the negative group.


(5) $$\text{HR}\;\left(\%\right)=\left[\frac{\left(A_{e}-A_{n}\right)}{\left(A_{p}-A_{n}\right)}\right]\times 100\%,$$

#### Cytocompatibility assay

NIH3T3 mouse embryonic fibroblasts were used to evaluate the biocompatibility of fiber sponges. Sponge samples were sterilized by UV light and immersed in DMEM solution for up to 24 h to obtain the extract solution from PU-ADP/Gel, PU-BDO/Gel, PU-DMPA/Gel and PU-TA/Gel (*n *=* *3). Cells were seeded into 48-well plates (2 × 10^4^ cells/well). About 400 μl of the extract solution was added into each well, followed by incubation at 37°C for 24 h. The absorbance was recorded at 450 nm; DMSO and DMEM served as positive and negative controls, respectively [42]. For cell proliferation assay, NIH3T3 fibroblasts were seeded onto the abovementioned fiber sponges and cultured for up to 1, 3 and 5 days. Cell proliferation was evaluated with cell counting kit-8 (CCK-8) and the absorbance was measured at 450 nm. Cell viability was evaluated by counting the number of live and dead cells. For live/dead assay, about 200 μl of the assay solution was added into each well and incubated for 20 min in dark. The cell on the samples was observed by using an inverted fluorescence microscope.

### *In vivo* wound healing assay

*In vivo* experiments were performed according to the guidelines of the Animal Committee of Tongji University, China. A total of 75 adult male SD rats (weight, 300–350 g) were divided into five groups, including control, A, B, C and D. Animals were anesthetized with 10% of chloral hydrate. Hairs were removed and full-thickness excisional defects (diameter, 13 mm) were created. Untreated wounds served as a control group. Wounds treated with PU-ADP/Gel, PU-BDO/Gel, PU-DMPA/Gel and PU-TA/Gel sponges were represented by groups A, B, C and D, respectively. At Days 3, 7 and 14, wounds were photographed and skin tissues were collected. Explants were fixed with paraformaldehyde for 24–48 h and were stained with Hematoxylin and Eosin (H&E) staining and Masson’s trichrome (MT) staining. Wound contraction (%) was calculated by Equation (6) [43]: where *A_i_* indicated the initial wound area, while *A_t_* represented the wound area at Days 3, 7 and 14, respectively.


(6) $$\mathrm{Wound}\;\mathrm{contraction}\;\left(\%\right)=\frac{A_{i}-A_{t}}{A_{i}}\;\times 100\%,\;$$

### Hemostatic assay *in vivo*

*In vivo* hemostatic performance of sponges was assessed with a total of 75 adult male SD rats, which were divided into five groups, including control, PU-ADP/Gel, PU-BDO/Gel, PU-DMPA/Gel and PU-TA/Gel (*n *=* *5). Rats were anesthetized with 10% chloral hydrate and shaved, after which, a transverse incision (length, 3 cm) was made in the middle of the upper abdomen to expose the liver. The left lobe of the liver was incised (1 × 0.5 cm), which led to the significant liver bleeding. Sponges were immediately applied to the incised liver and the amount of the bleeding and the hemostasis time were recorded. The experiment was repeated five times for each group. The gauze served as a control group. By the end of the experiments, animals were sacrificed under anesthesia.

### Statistical analysis

Each experiment had at least three replicates of data. All data were represented as mean ± standard deviation. Statistical analysis was performed with Origin 8.5 software. One-way ANOVA comparisons were performed with GraphPad Prism software. Probability values, such as *P <* *0.05, **P <* *0.01 and ***P < 0.001 were considered as statistically significant differences.

## Results and discussion

### Structural analysis

The PU was synthesized by using a two-step solution polymerization method and by using different types of chain extenders and reaction conditions. The synthesized polymers were named as PU-ADP, PU-BDO, PU-DMPA and PU-TA. The polymerization time for the PU-ADP and PU-BDO was 18 h, while for the PU-TA and PU-DMPA was 48 h and 120 h, respectively. The different polymerization time is ascribed to the different polymerization reaction activities of different chain extenders.

**Figure 1.**
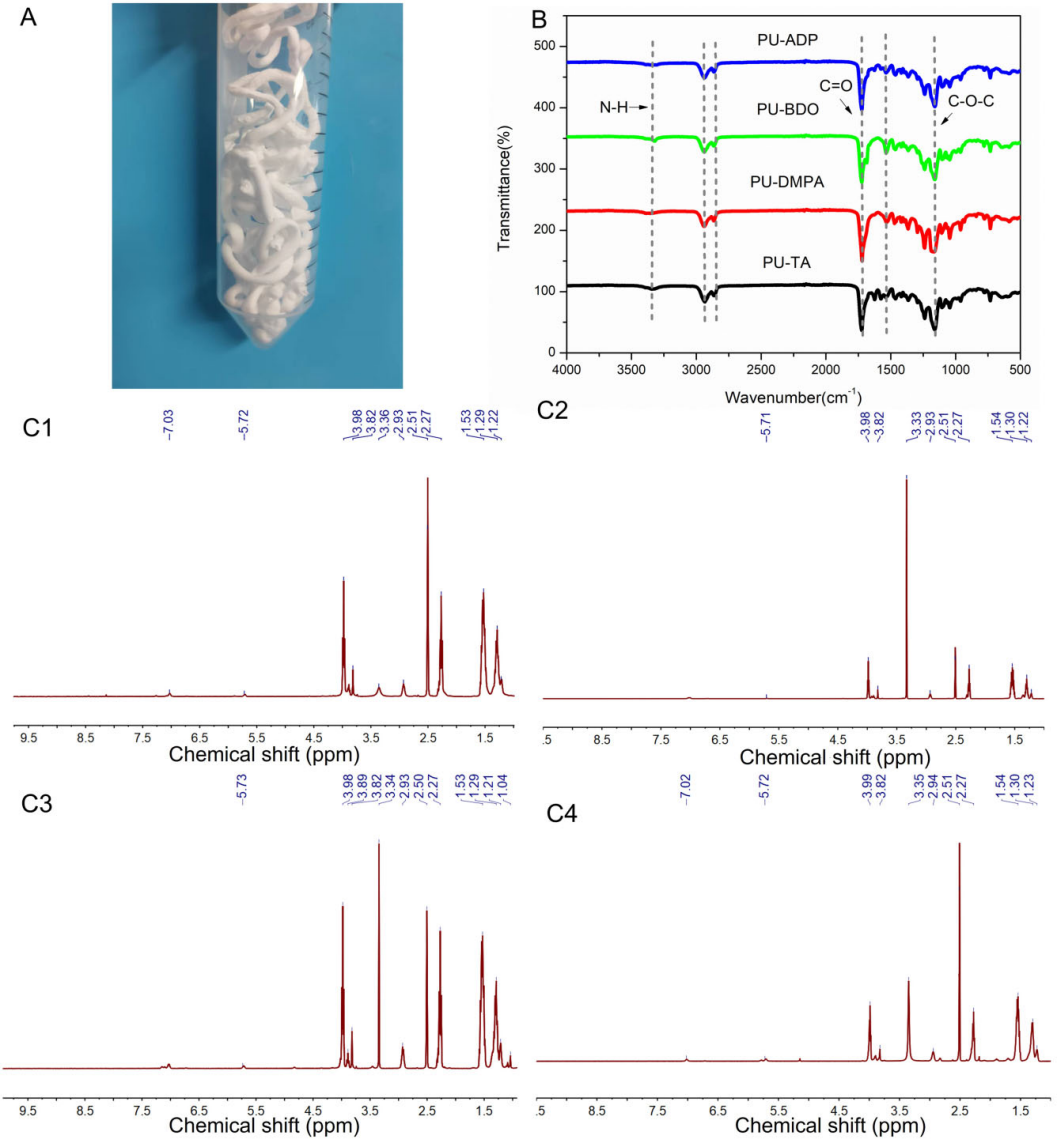
Structural analysis of PU. Photographs showing the synthetic PU-BDO (**A**). FTIR spectra of the synthesized PU (**B**). ^1^H-NMR spectra of PU-ADP (**C1**), PU-BDO (**C2**), PU-DMPA (**C3**) and PU-TA (**C4**).

The PU elastomers exhibited a noodle-like morphology (Fig. 1A). Structural analysis was performed by FT-IR (Fig. 1B). The bands appeared at 1723, 3350 and 1535 cm^−1^ were ascribed to the carbonyl (C=O) stretching, N–H bending and N–H stretching of the urethane group, respectively. The bands ascribed to the hydroxyl (–OH) groups of PCL diol and the isocyanate groups (–NCO) of HDI generally appear at 3500 and 2249 cm^−1^. Since the PU spectra did not exhibit these bands, it can be inferred that the hydroxyl (–OH) and isocyanate group (–NCO) were completely reacted.

Structural analysis was further carried out by ^1^H-NMR. Figure 1C1–C4 shows the spectra of PU-ADP, PU-BDO, PU-DMPA and PU-TA, respectively. The characteristic peaks of the HDI and PCL were appeared at 1.22, 1.3, 1.53, 2.27, 2.93, 3.82 and 3.98 ppm. The PU-ADP displayed a characteristics peak at 7.03 ppm, which was attributable to the ADP unit, the PU-BDO showed peaks at 1.53 and 3.98 ppm, which were ascribed to the BDO unit. On the other hand, 1.04 and 3.89 ppm peaks in the PU-DMPA spectra are ascribed to the DMPA unit, while a peak at 7.02 ppm in the PU-TA spectrum was the characteristic signal of the TA unit. These results indicated successful polymer synthesis with the chain extension.

### Morpholgical analysis of the fiber sponge

**Figure 2.**
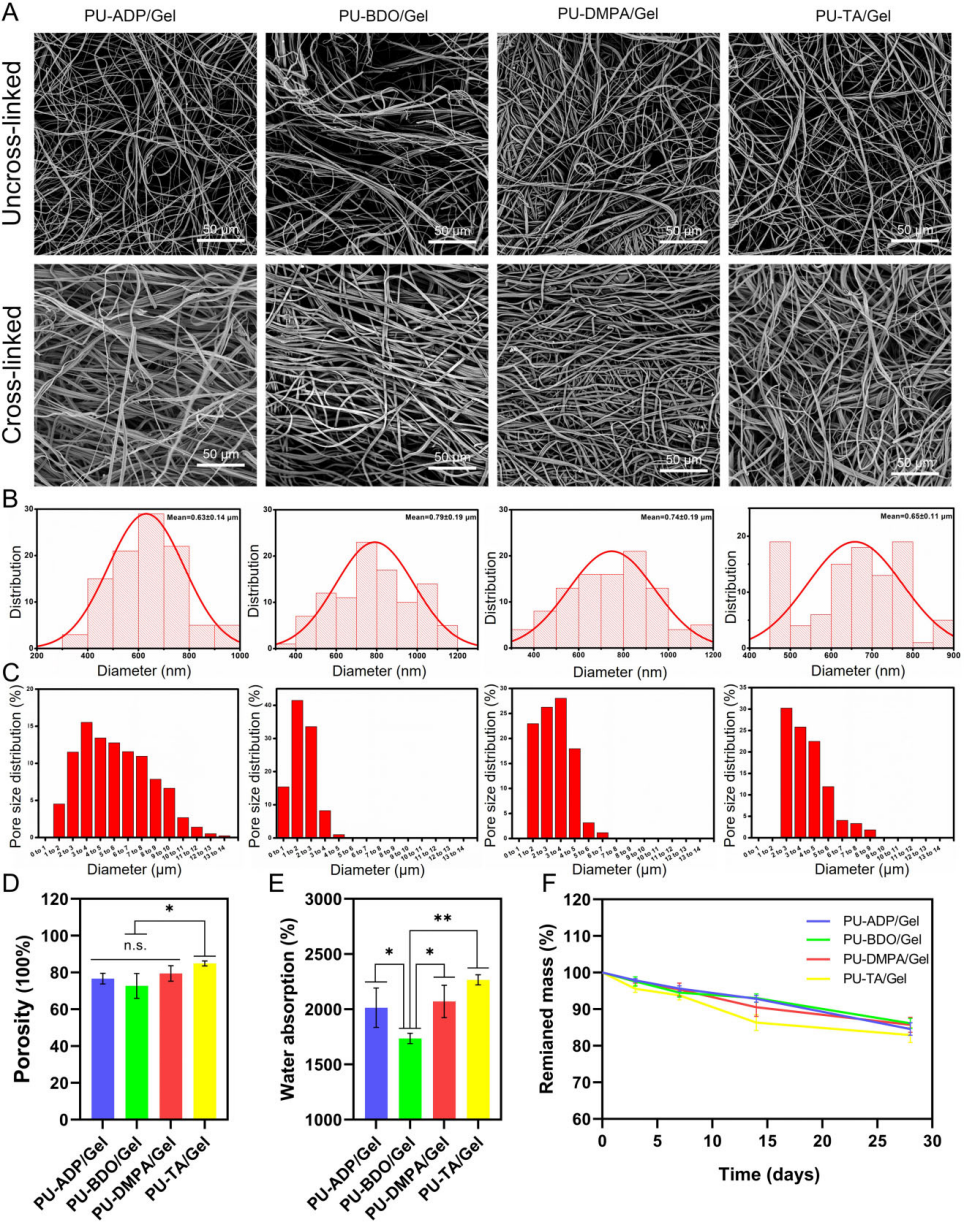
Characterization of fiber sponges. SEM micrographs of different types of fibers (**A**). Diameter (**B**) and pore size (**C**) distribution of cross-linked sponge. Porosity (**D**) and water absorption (**E**) of cross-linked sponge (*n *=* *3). Remained mass of different types of sponges (**F**). Error bars represent SD. *P <* *0.05, **P <* *0.01.

Electrospun sponges were analyzed by the SEM (Fig. 2A). All of the membranes exhibited smooth, uniform, and bead-free fibers. The distribution of fibers after cross-linking became dense. The average diameters of the fiber sponges were 0.63 ± 0.14, 0.79 ± 0.19, 0.74 ± 0.19 and 0.65 ± 0.11 μm for PU-ADP/Gel, PU-BDO/Gel, PU-DMPA/Gel and PU-TA/Gel, respectively (Fig. 2B). The porosity of sponges was assessed by nitrogen adsorption method. The pore diameter of PU-ADP/Gel, PU-BDO/Gel, PU-DMPA/Gel and PU-TA/Gel fiber sponges were concentrated in the range of 2–10, 0–4, 1–5 and 2–6 μm, respectively (Fig. 2C). These porous scaffolds may be beneficial for the growth and migration of cells as well as the diffusion and transport of the nutrients.

The porosity of fibrous scaffolds may also influence water absorption capacity [44]. As can be seen from Fig. 2D, all groups showed a porosity value of more than 70%; PU-TA/Gel sponge exhibited significantly higher porosity than that of the other groups, which also led to the higher water absorption. This higher water absorption may also have implications for the quick blood absorption and the cleaning of the wound exudate for the rapid hemostasis of the wound [45]. The water absorption rate of PU-ADP/Gel, PU-BDO/Gel, PU-DMPA/Gel and PU-TA/Gel sponge was found to be 2014 ± 178%, 1736 ± 46%, 2071 ± 146% and 2266 ± 46%, respectively (Fig. 2E). The higher water absorption rate of the PU-TA/Gel may be ascribed to the amino (–NH_2_) or carboxylic (–COOH) groups, which may facilitate water uptake.

For *in vitro* degradation, sponges were immersed in sterilized PBS at 37°C. As can be seen from Fig. 2F, fiber sponges lost their weight over time. While there was an insignificant difference among sponges in terms of the remaining weight both at Days 3 and 7, PU-TA/Gel showed fast degradation than that of the other sponges beyond Day 14. All sponges lost an average of 15% of their weight by Day 28. Minimal degradation of sponges may be ascribed to PU which is difficult to be hydrolyzed. Similarly, the poor degradation may also be attributable to the crosslinking with the GA vapors.

### Cytocompatibility

**Figure 3.**
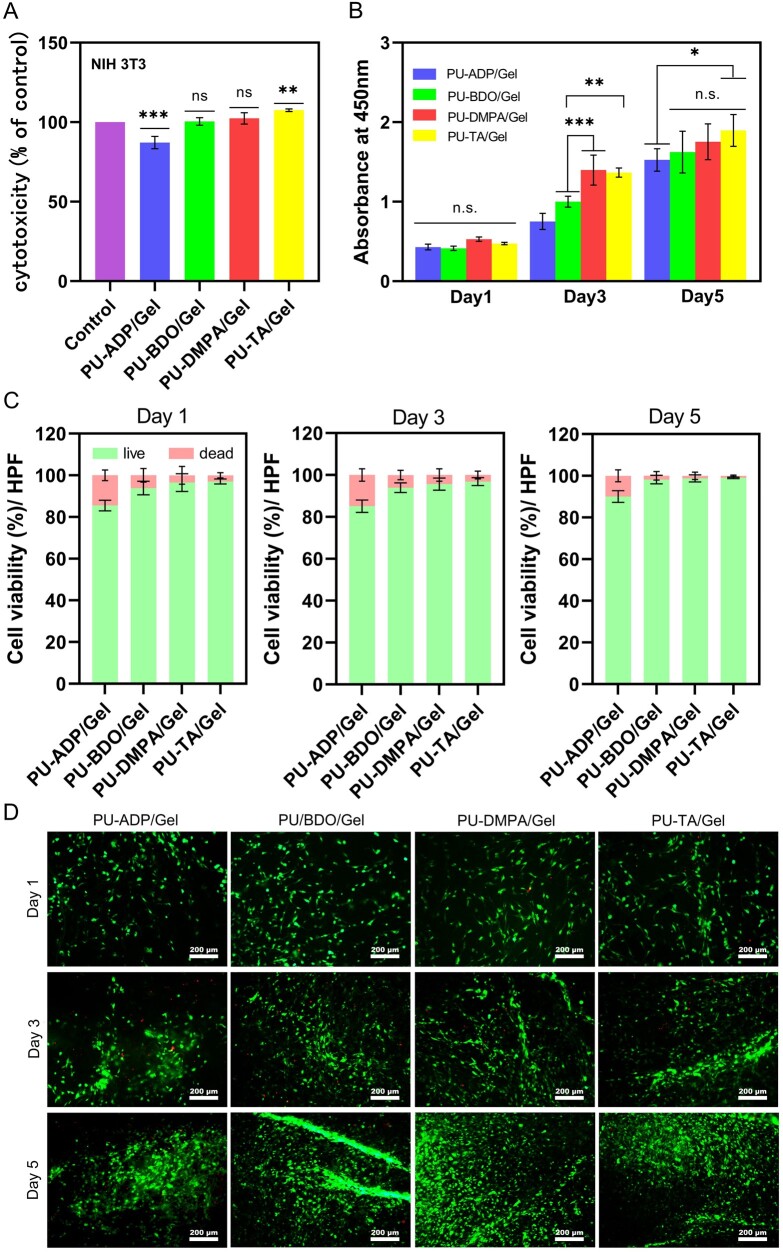
Cytocompatibility of scaffolds. Cytotoxicity of the extract solution collected from different types of fiber sponges cultured along with NIH3T3 fibroblasts for up to 24 h. DMEM served as a control group (**A**) (*n *=* *3). CCK-8 assay of NIH3T3 fibroblasts on sponges at Days 1, 3 and 5 (**B**) (*n *=* *3). Number of live and dead cells per high power field (hpf) at Days 1, 3 and 5 (**C**) (*n* = 3). Live/dead cell assay of cell-seeded scaffolds at different time points (**D**). Error bars represent SD. *P <* *0.05, **P < 0.01, ***P < 0.001.

NIH3T3 cells were cultured along with the extract solution obtained from different types of fiber sponges for up to 24 h and the cytotoxicity was assessed (Fig. 3A). The viability of DMEM was set at 100% as a control. After 24 h of cell culture, the cell viability in all of the groups was more than that of 85%. PU-TA/Gel exhibited significantly higher cell viability (108.38 ± 3.22% as compared to the control group), showing its potential benefit for cell proliferation.

The cytocompatibility of fibers was further assessed by a CCK-8 assay. As can be seen from Fig. 3B, the absorbance showed an increasing trend with an increase in the culture time, which indicated that the cells were continuously proliferating on the sponge. The absorbance values were found to be 1.5 ± 0.12, 1.6 ± 0.23, 1.75 ± 0.2 and 1.8 ± 0.18 for PU-ADP/Gel, PU-BDO/Gel, PU-DMPA/Gel and PU-TA/Gel sponges by Day 5. The PU-TA/Gel sponges exhibited significantly higher absorbance than that of the other groups, presumably due to its high porosity and hydrophilicity.

The cell viability was further ascertained from the fluorescence images obtained from the live/dead assay. Figure 3C showed an increasing number of live cells, and a gradual decrease of the dead cell over time, indicating that the sponges could promote cell proliferation. Live/dead staining showed strong green fluorescence, which indicated that the seeded cells on the sponges were predominately live (stained as green), while there were only a few numbers of dead cells (stained in the red color) (Fig. 3D). A good cell viability on sponges may be ascribed to their porous microstructure, which may be beneficial for cell adhesion.

### *In vitro* blood coagulation and hemolysis study

**Figure 4.**
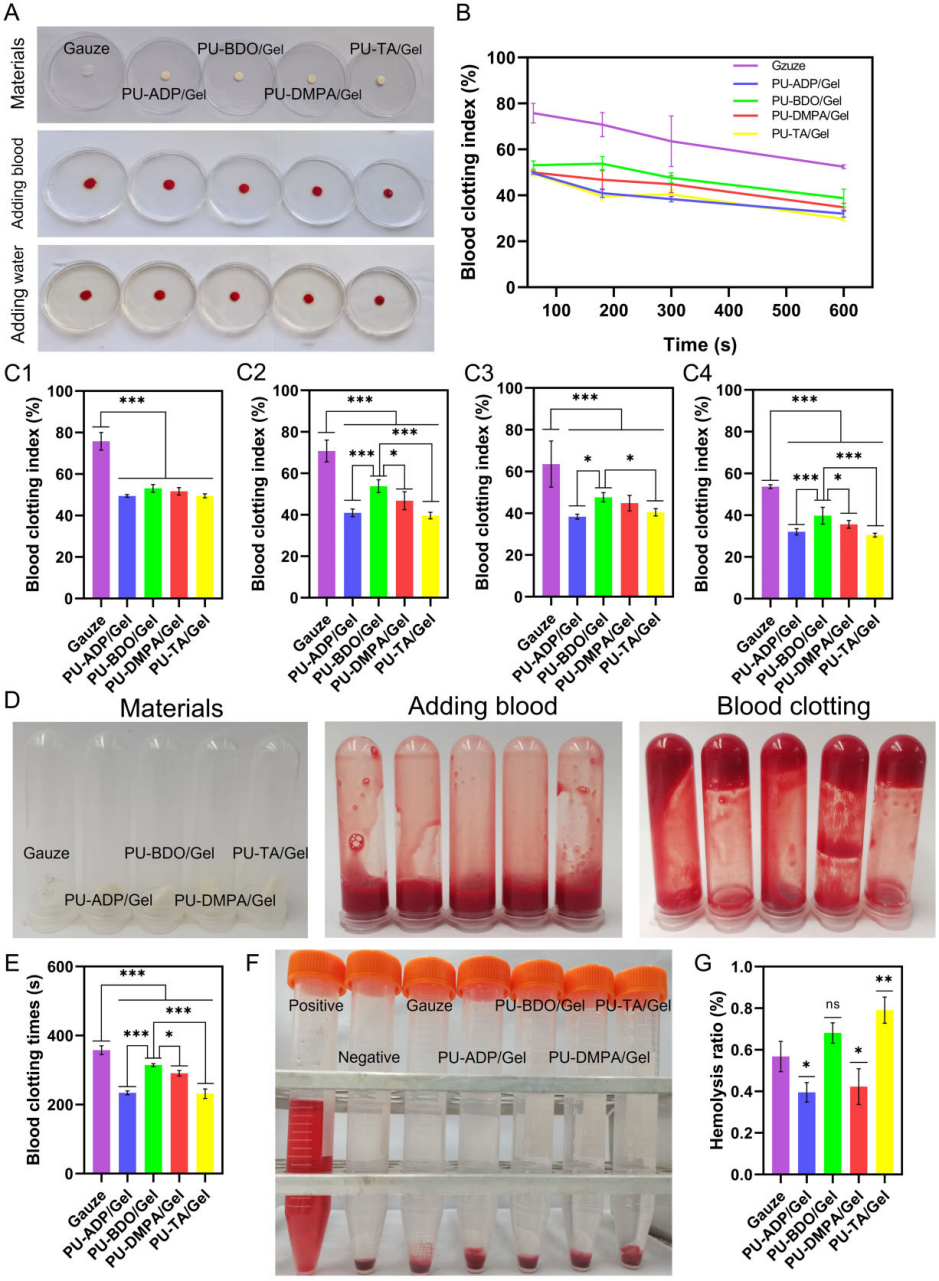
*In vitro* blood coagulation and hemocompatibility of the sponge. Blood clotting index (BCI) was measured to evaluate hemostatic ability of the sponges (**A**). Gauze served as a control. BCI of the sponges at different time points (**B**). BCI of different sponges at 1 (**C1**), 3 (**C2**), 5 (**C3**), and 10 (**C4**) min (*n* = 6). The photographs of the blood clotting process after the application of sponges (**D**). Blood clotting time (**E**) (*n* = 4). Photographs of hemolysis assays, DI water (positive control) and normal saline (negative control) (**F**). Hemolysis ratio (*n* = 4) (**G**). Error bars represent SD. *P < 0.05, **P < 0.01, ***P < 0.001.

Hemostatic ability of sponges was evaluated by a whole blood clotting assay. As can be seen from Fig. 4A, the gauze group showed an evident increase in the color of the water. On the other hand, in the sponge group, the color was lighter than that of the gauze, which indicates that the blood coagulation effect of the sponge was higher than that of the gauze. The BCI value of the material is determined by the absorbance of the hemoglobin from the uncoagulated erythrocytes; a high value of BCI indicates poor blood clotting ability [46, 47]. As can be seen from Fig. 4B, BCI value decreased with an increase in the incubation time. The PU-TA/Gel sponge showed significantly less BCI values than that of the other sponges. The BCI values were found to be 53.71 ± 0.88%, 32.05 ± 1.47%, 39.75 ± 4.06%, 35.64 ± 1.84% and 30.42 ± 0.91% for gauze, PU-ADP/Gel, PU-BDO/Gel, PU-DMPA/Gel and PU-TA/Gel sponges respectively at 10 min (Fig. 4C). Sponges showed less BCI values than that of the gauze; PU-TA/Gel showed significantly less value of the BCI, which indicated its good blood clotting capacity.

*In vitro* hemostatic effects of the sponges were assessed by measuring blood clotting time. As can be seen from Fig. 4D, sponges fostered blood coagulation and clotting. All fiber sponges exhibited short blood clotting time than that of the gauze group. The blood clotting time of PU-ADP/Gel, PU-BDO/Gel, PU-DMPA/Gel and PU-TA/Gel was 234 ± 5, 314 ± 4, 290 ± 7 and 231 ± 13 s, respectively, while that of the gauze group was 357 ± 12 s (Fig. 4E). Fast blood clotting is ascribed to the fibrous structure of sponges than that of the gauze. Moreover, the short blood clotting time of PU-TA/Gel sponge may be ascribed to the antifibrinolytic effect of TA, which may inhibit an interaction between plasminogen and plasmin, thereby inducing rapid clot formation [48].

Hemocompatibility of fiber sponges was studied by hemolysis assay. Hemolysis ratio of all fiber sponges was <5%, therefore in agreement with the acceptable standard of below 5% of hemolysis (Fig. 4F–G). Moreover, the good hemostatic ability of fiber sponges is ascribed to the gelatin, which can induce platelet adhesion, activation and aggregation. Good hemostatic ability of PU-TA/Gel may also be attributed to the synergistic effect of TA and Gel. The TA may strongly adsorb to the lysine binding site of the fibrin affinity site on plasmin and plasminogen, thereby suppressing the binding of plasmin and plasminogen to fibrin and inhibiting plasmin-induced fibrin decomposition.

### Hemocompatibility

**Figure 5.**
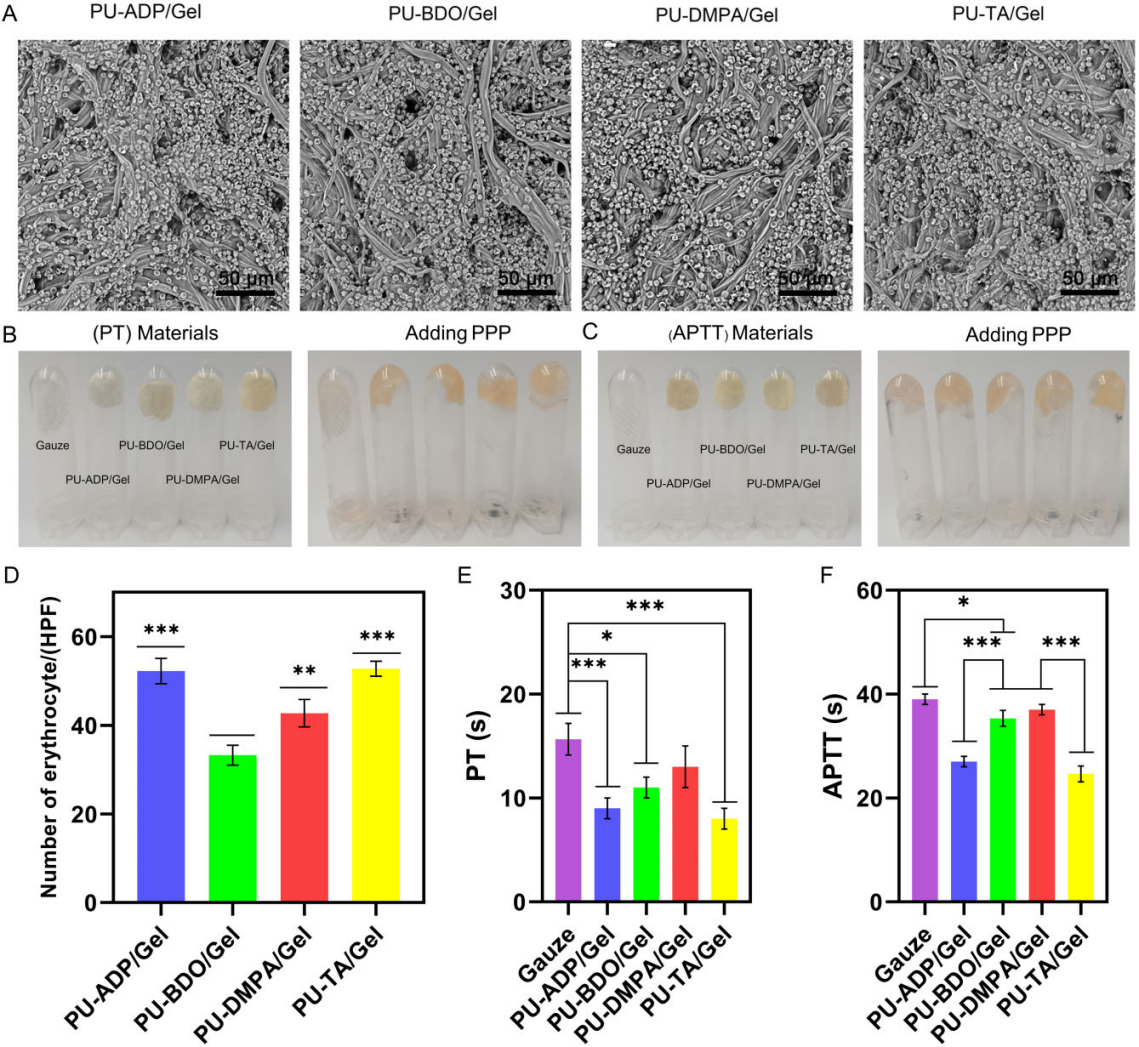
SEM micrographs showing the adhesion of erythrocytes on fiber sponges (**A**). photographs obtained from PT (**B**) and APTT (**C**) assay. Number of erythrocytes per hpf (**D**) (*n *=* *4). PT (**E**) and APTT (**F**) of fiber sponges as well as gauze (*n *=* *3). Error bars represent SD. *P < 0.05, **P < 0.01, ***P < 0.001.

Hemocompatibility of fiber sponges was next assessed. of the adhesion of erythrocytes to the surface of fibers is shown in Fig. 5A. As can be seen from this figure, a lot of erythrocytes were adhered to the fibers, which may be ascribed to an electrostatic interaction between positively charged Gel and negatively charged erythrocytes. Moreover, as can be seen from Fig. 5D, the density of the erythrocytes was significantly higher in fiber sponges than that of the gauze group; PU-TA/Gel showed.

The coagulation pathway was next ascertained by measuring PT and APTT (Fig. 5B and C). Except for PU-DMPA/Gel fibers, all fiber sponges manifested significantly lower PT values than that of the gauze (Fig. 5E). PU-TA/Gel sponges fibers displayed significantly less PT values as compared to all groups, which could effectively activate the extrinsic coagulation pathway. Similarly, PU-TA/Gel exhibited significantly smaller value for APTT as compared to other groups, which indicated obviously higher activation of an intrinsic coagulation pathway (Fig. 5F). These results indicated that PU-TA/Gel sponge can activate both the extrinsic and intrinsic coagulation pathways, which may have implications for the hemocompatibility of fibers.

### Hemostatic assay of fiber sponges in a liver model in rats

**Figure 6.**
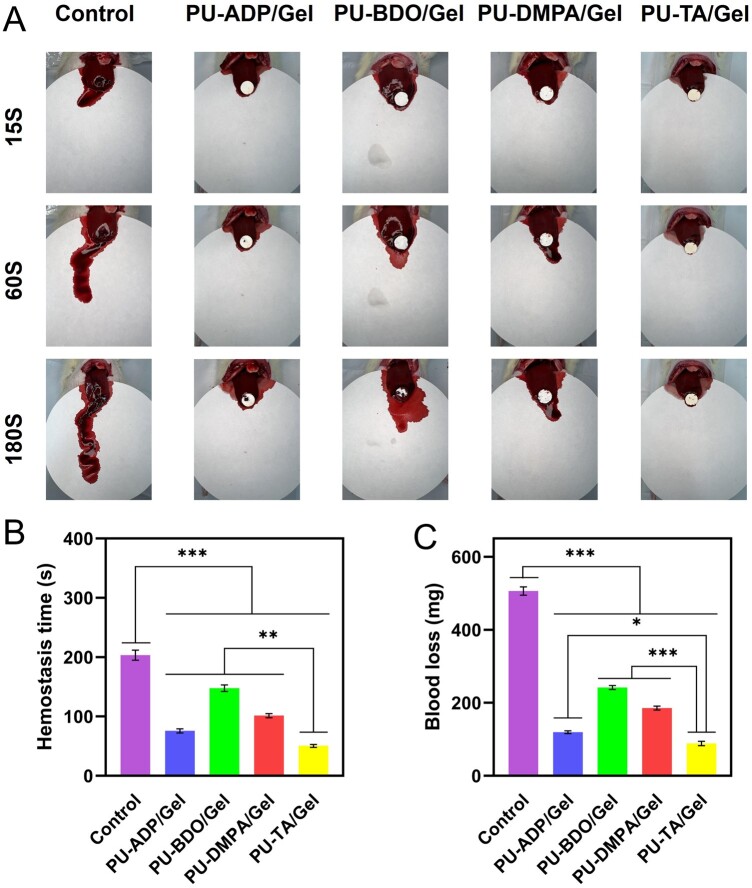
Optical photographs showing the application of different samples for the hemostatic assay of different types of materials in a liver injury model in rats (**A**). Hemostasis time (**B**) (*n *=* *5). Blood loss (**C**) (*n *=* *5). Error bars represent SD. *P <* *0.05, **P <* *0.01, ***P < 0.001.

*In vivo* hemostatic effect of gauze as well as different types of fiber sponges were assessed in a liver trauma model in rats (Fig. 6A). The control group exhibited hemostasis time of 203 ± 9 s. On the other hand, hemostasis time was found to be 76 ± 4, 148 ± 6, 101 ± 4 and 50 ± 3 s for PU-ADP/Gel, PU-BDO/Gel, PU-DMPA/Gel and PU-TA/Gel sponges, respectively (Fig. 6B). As can be seen from these results, PU-TA/Gel-based fiber sponges required significantly less time for hemostasis time, which was also consistent with the amount of blood loss. The control group showed a significantly higher content of blood loss (506 ± 11 mg) than the other groups. The amount of blood loss was 119 ± 4, 241 ± 6, 185 ± 5 and 89 ± 6 mg for PU-ADP/Gel, PU-BDO/Gel, PU-DMPA/Gel and PU-TA/Gel sponges, respectively. (*P < 0.05) (Fig. 6C). Taken together, PU-TA/Gel sponges had the best hemostatic effect as compared to the other groups in a liver trauma model in rats, which may be partly attributed to the good absorption of fluid.

### Wound healing

**Figure 7.**
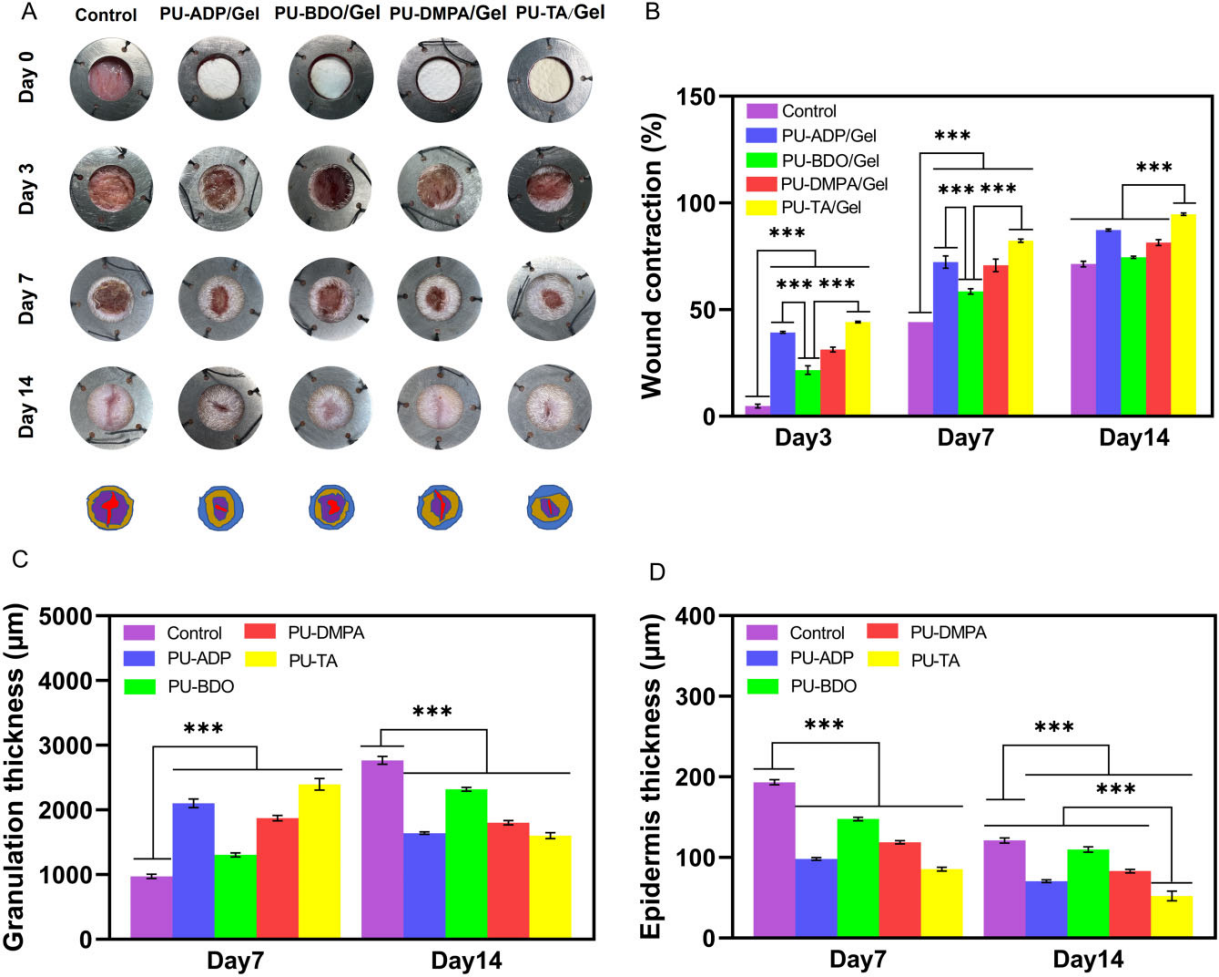
Photographs showing the wound size at Days 0, 3, 7 and 14 after the application of different types of sponges (**A**). Untreated groups served as controls. Wound contraction at different time points (**B**) (*n *=* *5). The thickness of granulation tissues (**C**) and epidermis (**D**) (*n* = 5). Error bars represent SD. ***P < 0.001.

The process of wound healing is complex, including wound hemostasis, cell proliferation, cell migration, inflammatory response, tissue regeneration and so on [49]. The effectiveness of different types of fiber sponges was assessed in full-thickness excisional defect models in rats. As can be seen from Fig. 7A and B, the contraction was significantly higher in the groups treated with the PU-TA/Gel than that of the control as well as other groups both at Days 3 and 7, which further increased for up to 95% by Day 14. These results revealed that the PU-TA/Gel group had a better wound healing effect than that of the other groups.

**Figure 8.**
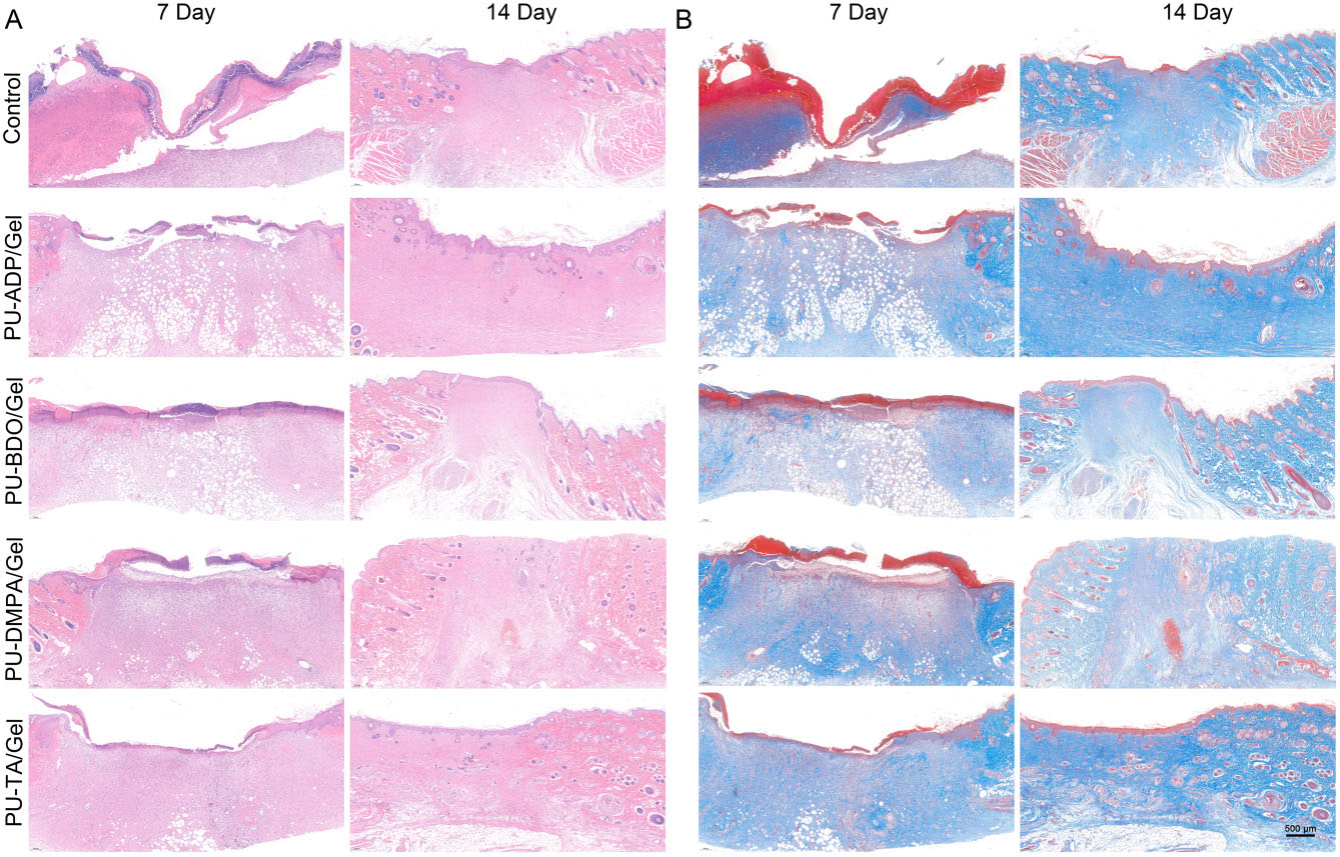
H&E (**A**) and MT (**B**) staining of full thickness excisional defects treated with different types of sponges at Days 7 and 14.

H&E staining and MT stainings were performed on wound tissues to evaluate the wound-healing effect of sponges (Fig. 8A and B). H&E staining showed the formation of granulation tissues during wound healing, which had a clear boundary with the surrounding normal mature skin tissue. The extent of granulation tissues was decreased over time. By Day 14, the groups treated with sponges manifested more wound healing than that of the untreated control group. Intriguingly, PU-TA/Gel group showed significantly less granulation tissues than that of the other groups, which showed its better wound healing potential.

It is well-established that the granulation tissues play varying roles during the different stages of wound healing. In the initial stage, new capillaries in granulation tissue may be helpful to remove injured and necrotic tissues as well as increase the diffusion of oxygen and the transport of nutrients, thereby helping the wound healing. On the other hand, in the later stages, the diminished formation of granulation tissues may be helpful for collagen deposition and ECM remodeling. As mentioned above, the content of granulation tissues was obviously higher in PU-TA/Gel-treated groups than that of the other groups, which may be helpful to improve neovascularization as well as promote tissue repair. In contrast, by Day 14, the thickness of granulation tissue in the PU-TA/Gel group was decreased and was found to be substantially less than that of the other groups, which revealed that the granulation tissues were successfully remodeled and evolved into normal tissues (Fig. 7C).

The thickness of the epidermis is closely related to tissue healing. However, in comparison with the healthy tissues, an increase in the epidermis thickness may lead to an increase in the scar tissue due to inflammatory tissue stimulation and abnormal differentiation. PU-TA/Gel group manifested significantly less thickness of the epidermis than that of the control group as well as other groups both at Days 7 and 14 (Fig. 7D). By Day 14, the thickness of epidermis in PU-TA/Gel-treated groups was comparable to the normal tissue, indicating the superiority of these sponges over other to promote tissue repair and accelerate wound healing.

This study has also some limitations: (i) since PU is chemically synthesized, batch-to-batch differences may affect the properties of different types of sponges. (ii) The fabrication process of sponges also need further optimization to afford mass produced fibers. Nevertheless, the good hemostatic ability and hemocompatibility of these modular PU-based materials may be helpful to afford functional scaffold materials for different types of biomedical applications.

## Conclusions

Taken together, we synthesized different types of PU by varying the types of chain extenders. These polymers were next blended along with Gel to afford fiber sponges, which afforded smooth and uniform fibers with sufficient porosity. *In vitro* hemocompatibility and hemostatic assays revealed promising potential of PU-TA/Gel-based sponges than that of the other groups, which was ascribed to the synergistic effect of TA and Gel. PU-TA/Gel-based sponges were also found to be better than that of the gauze and other groups for hemostatsis in a liver trauma model in rats, which led to the formation of stable blood clots along with less blood clotting time. More importantly, PU-TA/Gel fibers also modulated granulation tissue formation at different stages of wound healing and afforded fast wound healing than that of the other groups.
